# Gastric Bezoar Composed of Intertwined Mobile Phone Charging Cables in a Young Adult With Autism Spectrum Disorder and Pica: A Case Report

**DOI:** 10.7759/cureus.117446

**Published:** 2026-10-04

**Authors:** Sergio Miguel Suárez Fuentes, Mauro A Cardenas, Luis F Hernández González, José J Cervantes De Anda, Soledad Merced Castro Olmedo, Arym P Preza Estrada, Hansel F Ramos Ramírez, Nora Lis Flores-Olmos, Patricia G Arriaga Felipe

**Affiliations:** 1 Plastic and Reconstructive Surgery, Hospital Regional Valentín Gómez Farías, Instituto de Seguridad y Servicios Sociales de los Trabajadores del Estado (ISSSTE), Zapopan, MEX; 2 Surgery, Hospital General Presidente Lazaro Cardenas del Rio, Instituto de Seguridad y Servicios Sociales de los Trabajadores del Estado (ISSSTE), Chihuahua, MEX; 3 General Surgery, Hospital Regional Valentín Gómez Farías, Instituto de Seguridad y Servicios Sociales de los Trabajadores del Estado (ISSSTE), Zapopan, MEX

**Keywords:** autism spectrum disorder, charging cable, endoscopy, foreign-body ingestion, gastric bezoar, gastrotomy, pica, recurrent ingestion, surgery

## Abstract

Bezoars composed of nonfood foreign material are uncommon and may pose substantial diagnostic and therapeutic challenges, particularly in patients with pica and neurodevelopmental disorders. We report a 22-year-old man with autism spectrum disorder and a remote history of laparotomy for ingestion of batteries and magnets who presented for recurrence of absence seizures. A radiopaque intragastric foreign-body conglomerate was identified incidentally on radiography. Plain abdominal imaging demonstrated numerous linear, serpiginous radiopaque structures conforming to the gastric contour without evidence of bowel obstruction or perforation. Upper endoscopy showed the gastric cavity to be almost completely occupied by a dense network of intertwined cables, with associated erythematous and erosive gastropathy. Endoscopic retrieval with a metallic snare was unsuccessful because of the volume and entanglement of the material. The patient subsequently underwent open supraumbilical laparotomy and anterior gastrotomy, allowing en bloc removal of a bezoar composed predominantly of mobile phone charging cables. The gastrotomy was closed in two layers, and a closed-suction drain was placed. Recovery was uncomplicated; oral intake was progressively advanced, the drain was removed at discharge, and follow-up at one and two weeks showed adequate wound healing and no gastrointestinal symptoms. Psychiatry established a diagnosis of pica and arranged outpatient follow-up. This case highlights the potential for clinically silent, prolonged accumulation of unusual foreign material in the stomach and the importance of integrating radiographic, endoscopic, surgical, and psychiatric management when endoscopic extraction is not feasible.

## Introduction

Bezoars are compact intraluminal aggregates of indigestible material that most commonly form in the stomach. Classical categories include phytobezoars, trichobezoars, pharmacobezoars, and lactobezoars, although bezoars composed of miscellaneous nonfood foreign material are increasingly recognized in the setting of pica and recurrent intentional ingestion [1,2]. Their clinical expression depends on composition, size, location, and duration; patients may remain minimally symptomatic for prolonged periods or may present with pain, vomiting, gastric outlet obstruction, mucosal ulceration, bleeding, perforation, or distal intestinal obstruction [1].

Pica is characterized by persistent ingestion of nonnutritive, nonfood substances and is disproportionately encountered in individuals with neurodevelopmental disorders. In a large case-control study of young children, pica was substantially more prevalent among those with autism spectrum disorder (ASD) than among population controls, underscoring the importance of considering recurrent ingestion in this population [3]. Repeated ingestion can lead not only to isolated foreign bodies but also to conglomerates that become mechanically difficult to retrieve, particularly when long, flexible objects become intertwined.

Plain radiography is useful when radiopaque objects are suspected, while endoscopy provides direct visualization and may permit therapeutic extraction. Guidelines for ingested upper gastrointestinal foreign bodies emphasize that management should be individualized according to object type, size, shape, location, symptoms, and evidence of complications; computed tomography (CT) is particularly useful when perforation or another complication is suspected [4,5]. When a bulky or tightly entangled foreign-body mass cannot be safely removed endoscopically, operative gastrotomy remains a definitive option.

We report an unusual gastric bezoar composed predominantly of intertwined mobile phone charging cables in a young adult with ASD and psychiatrist-confirmed pica. The case is notable for its largely silent gastrointestinal course, incidental radiographic discovery, failed endoscopic retrieval, and successful en bloc surgical extraction.

## Case presentation

A 22-year-old man with ASD diagnosed at four years of age was admitted after recurrence of an absence seizure following several years without reported episodes. His relevant history included two absence seizures during childhood and a laparotomy seven years earlier for ingestion of foreign bodies consisting of batteries and magnets. He was not taking chronic medication before this admission and had no known drug allergies. He denied alcohol, tobacco, and recreational drug use. During the initial medical evaluation, levetiracetam was started for the recurrent seizure episode. No metabolic or electrolyte abnormality was identified during the admission, and because the patient had not been receiving chronic antiseizure therapy after several seizure-free years, impaired absorption of an antiepileptic medication was not considered a plausible mechanism. The seizure recurrence and the gastric bezoar were therefore regarded as coincidental findings.

A radiograph obtained during the admission unexpectedly demonstrated a foreign-body conglomerate projected over the stomach, prompting evaluation by general surgery. Family members reported that ingestion of the material could have occurred months earlier. Despite the substantial intragastric burden, the patient did not report abdominal pain at surgical assessment. He was afebrile and hemodynamically stable apart from tachycardia at admission. The abdomen was soft, non-distended on examination, and non-tender to superficial and deep palpation, with no rebound or other signs of peritoneal irritation. Routine preoperative laboratory testing did not reveal leukocytosis, renal dysfunction, hepatobiliary enzyme elevation, or pancreatic enzyme elevation requiring a change in surgical management (Table 1).

**Table 1:** Relevant laboratory findings at admission Reference ranges shown are conventional adult reference intervals; institutional ranges may vary slightly. AST: aspartate aminotransferase; ALT: alanine aminotransferase; INR: international normalized ratio

Laboratory parameter	Result	Unit	Reference range
Hemoglobin	16.9	g/dL	13.5-17.5
Leukocyte count	9.77	×10³/µL	4.0-10.0
Neutrophils	71	%	40-75
Platelet count	306	×10³/µL	150-450
Creatinine	0.83	mg/dL	0.7-1.3
Total bilirubin	0.23	mg/dL	0.2-1.2
Direct bilirubin	0.09	mg/dL	0.0-0.3
Indirect bilirubin	0.14	mg/dL	0.2-0.9
AST	14	U/L	10-40
ALT	11	U/L	7-56
Amylase	53	U/L	30-110
Lipase	38.7	U/L	13-60
Lactate	1.2	mmol/L	0.5-2.0
INR	1.02	-	0.8-1.2

A formal radiologic interpretation of the abdominal radiograph was obtained after the initial surgical assessment. It described numerous linear and serpiginous radiopaque structures in the epigastrium conforming to the morphology of the stomach, compatible with a bezoar. The bowel gas pattern showed no dilated loops or radiographic findings suggesting obstruction, and no free intraperitoneal gas was reported. The striking gastric-shaped configuration of the radiopaque material is shown in Figure 1.

**Figure 1:**
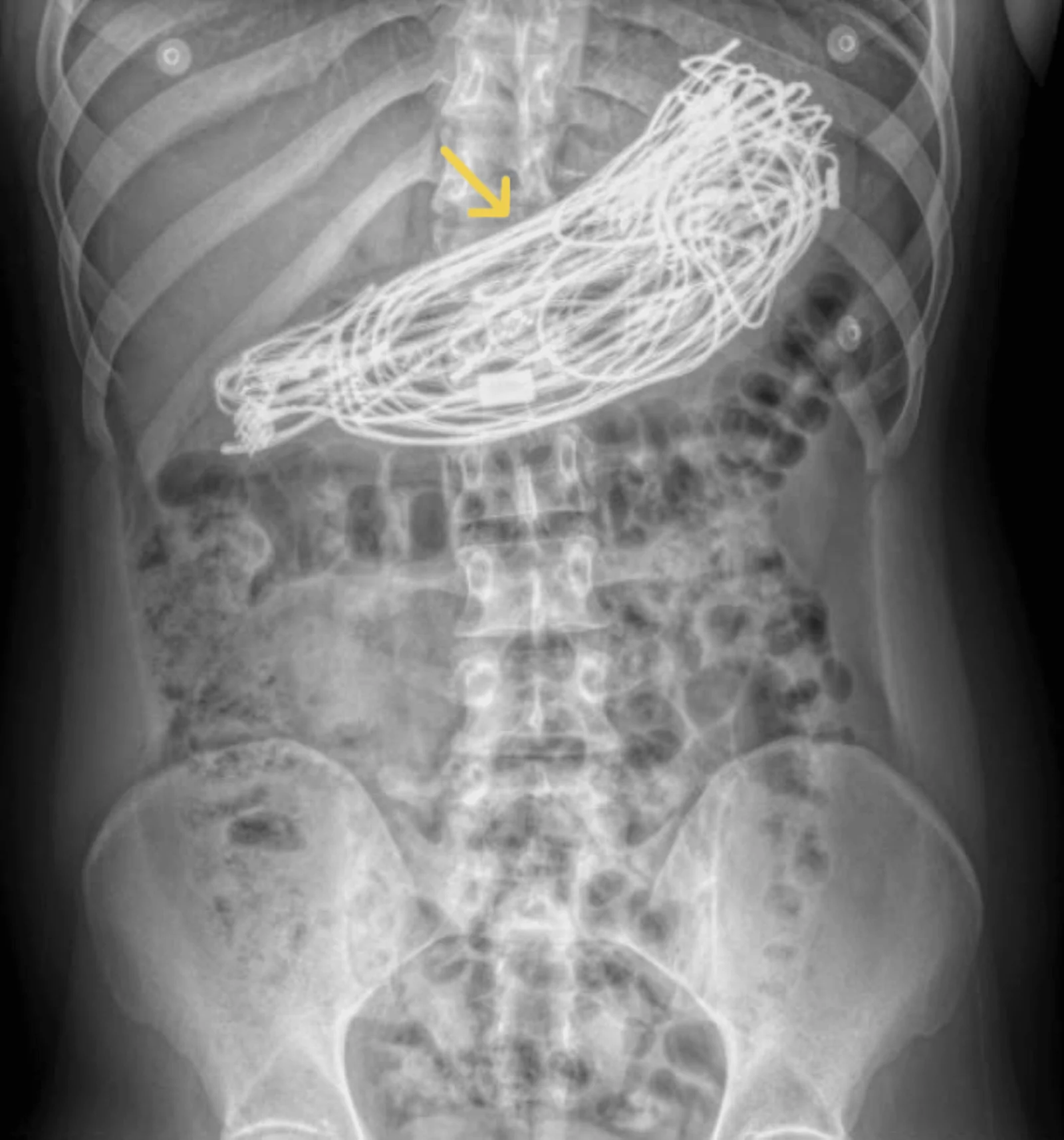
Plain abdominal radiograph demonstrating a gastric-shaped conglomerate of radiopaque cables Supine anteroposterior abdominal radiograph showing numerous linear and serpiginous radiopaque structures projected over the epigastrium and conforming to the gastric contour, producing the appearance of a gastric foreign-body bezoar. The arrow indicates the radiopaque conglomerate of intertwined cables conforming to the gastric contour. The formal radiology report described no dilated bowel loops or radiographic evidence of free intraperitoneal gas.

Upper gastrointestinal endoscopy was performed three days after the initial presentation. The esophageal mucosa was normal, and the gastroesophageal junction was competent on retroflexion. The gastric cavity was occupied almost completely by inorganic material forming a dense network of intertwined cables. In the evaluable gastric mucosa, erythematous gastropathy was present, and the antrum demonstrated multiple erythematous pseudopolypoid areas with superficial erosion but no active bleeding. The pylorus was patent, and the bulb and second portion of the duodenum appeared normal. Several cable segments and a connector tip consistent with a mobile-device charging cable were directly visualized (Figure 2). A limited therapeutic attempt was made using a metallic snare. No overtube or transparent hood was used. The conglomerate remained completely fixed and could not be mobilized even minimally; therefore, transesophageal withdrawal was not attempted. Further traction was promptly abandoned because continued manipulation was considered to carry an unacceptable risk of gastric or esophageal mucosal injury and perforation.

**Figure 2:**
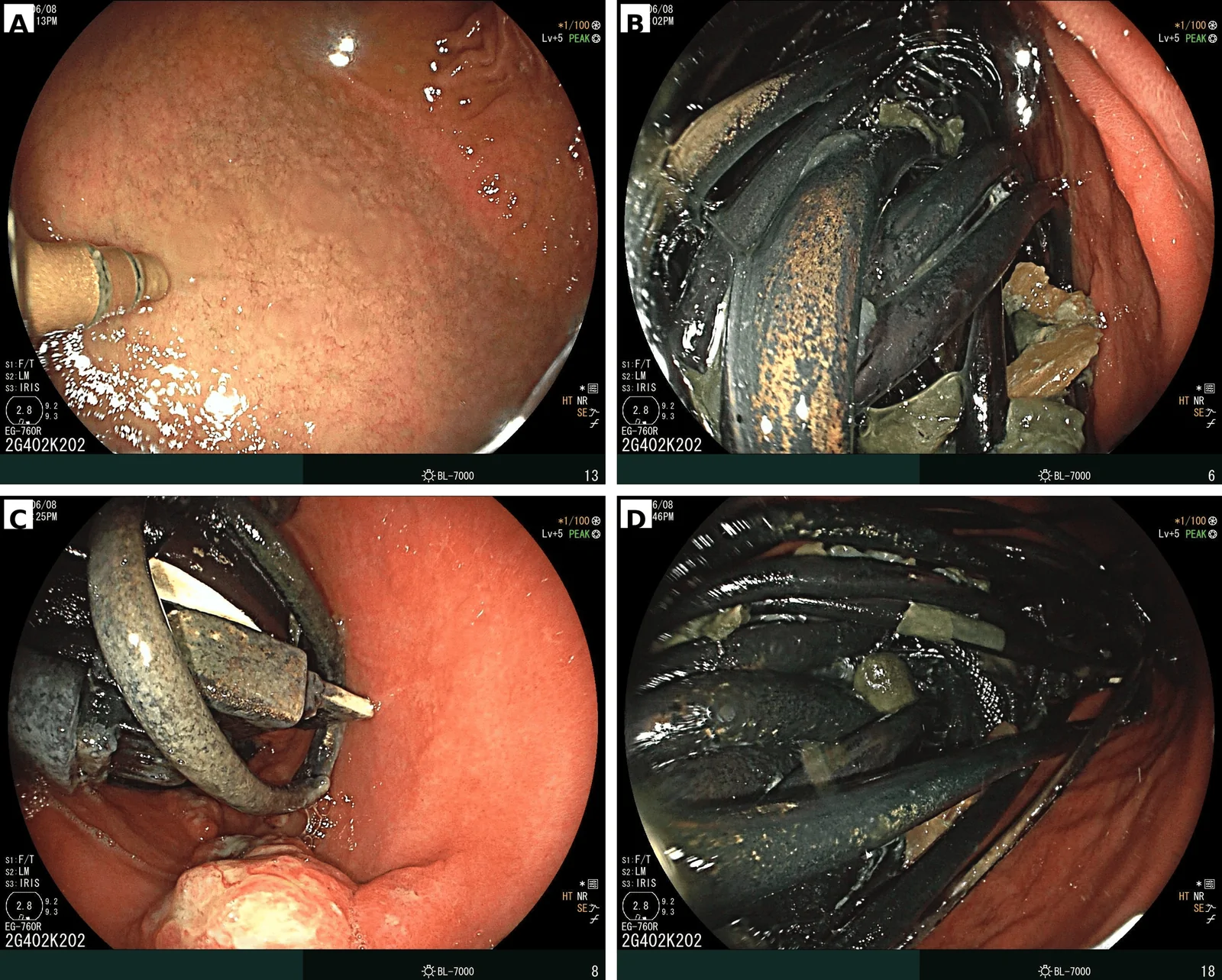
Endoscopic findings of the gastric cable bezoar and associated mucosal injury Upper gastrointestinal endoscopy performed before operative management. (A) Erythematous and erosive antral mucosal changes associated with chronic contact from the retained foreign material. (B) Dense network of intertwined cables occupying the gastric lumen. (C) Close endoscopic view showing a cable connector tip, supporting the identification of the material as mobile-device charging cables. (D) Additional view demonstrating the compact, intertwined configuration of the intragastric cable mass.

The patient was maintained fasting and referred for operative removal.

That night, under general anesthesia, an open supraumbilical midline laparotomy was performed. The stomach was markedly distended and firm because of the intraluminal mass. A longitudinal anterior gastrotomy of approximately 7 cm was created in the antropyloric region and controlled with atraumatic Babcock clamps. A large conglomerate of multiple intertwined electrical charging cables occupied nearly the entire gastric lumen. The mass was carefully mobilized and removed en bloc through the gastrotomy, avoiding fragmentation and additional gastric-wall trauma. The lumen was then inspected visually and palpated to exclude residual foreign material. The sequential operative findings are shown in Figure 3.

**Figure 3:**
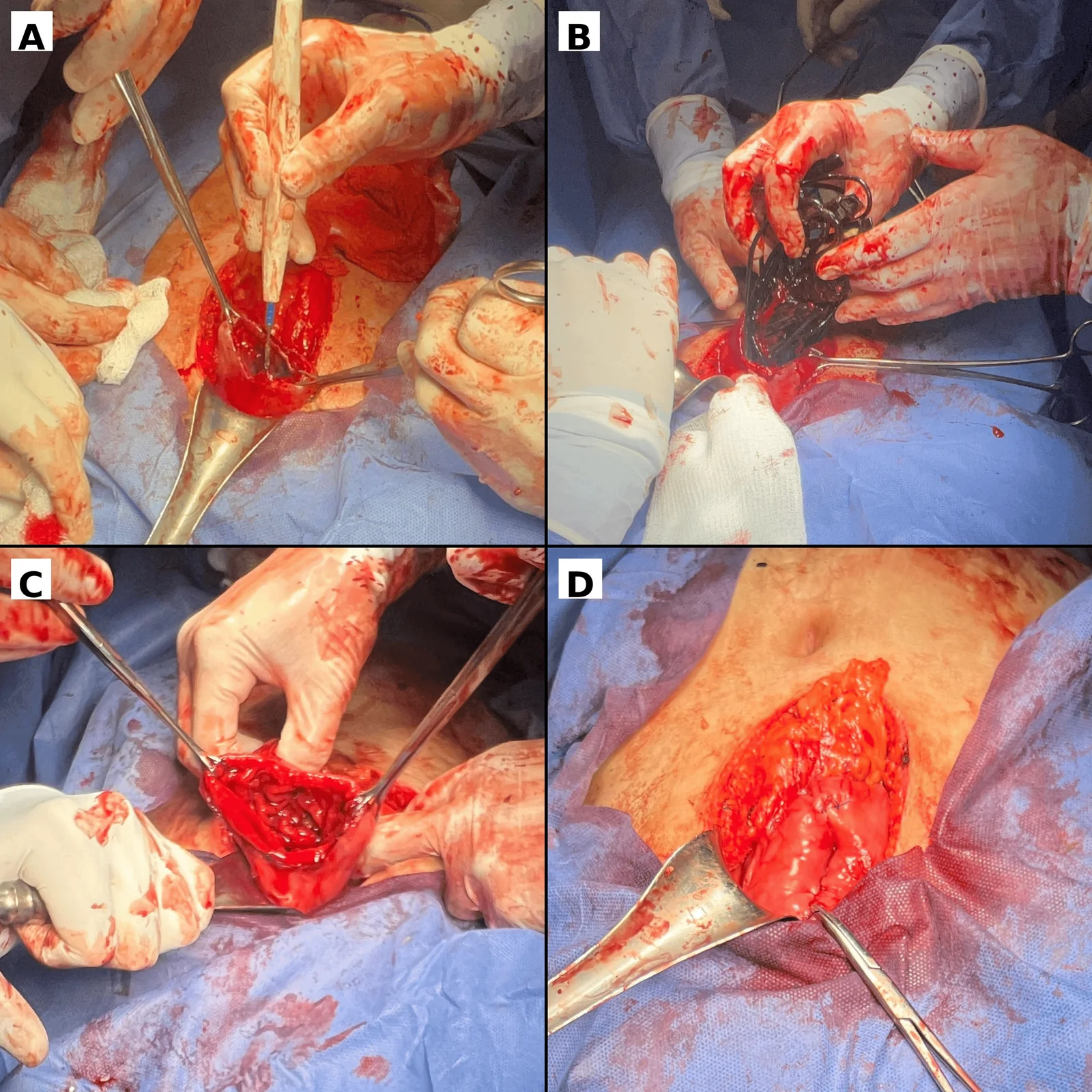
Sequential operative removal of the gastric cable bezoar through anterior gastrotomy Intraoperative sequence during open surgical management. (A) Anterior gastrotomy providing direct access to the gastric lumen. (B) En bloc extraction of the intertwined cable bezoar through the gastrotomy. (C) Gastric lumen after removal, with the distal lumen toward the antrum free of the foreign-body mass. (D) Final appearance after two-layer closure of the gastrotomy.

The gastrotomy was closed in two layers using 0 polypropylene: a first layer approximating the gastrotomy edges and a second continuous seromuscular inverting Lembert layer. The use of polypropylene reflected the operating surgeon's technical preference rather than a presumed advantage over delayed-absorbable suture material. The abdominal cavity was irrigated with isotonic saline, and a closed-suction Blake drain was positioned adjacent to the gastrotomy. The abdominal wall was closed in layers and the skin with metallic staples. No intraoperative complication was documented. Gross inspection of the extracted material identified seven intertwined cables, with approximate individual lengths ranging from 30 cm to 100 cm and an estimated combined weight of approximately 200 g. Most were mobile phone charging cables, while at least one segment appeared compatible with an auxiliary audio cable (Figure 4).

**Figure 4:**
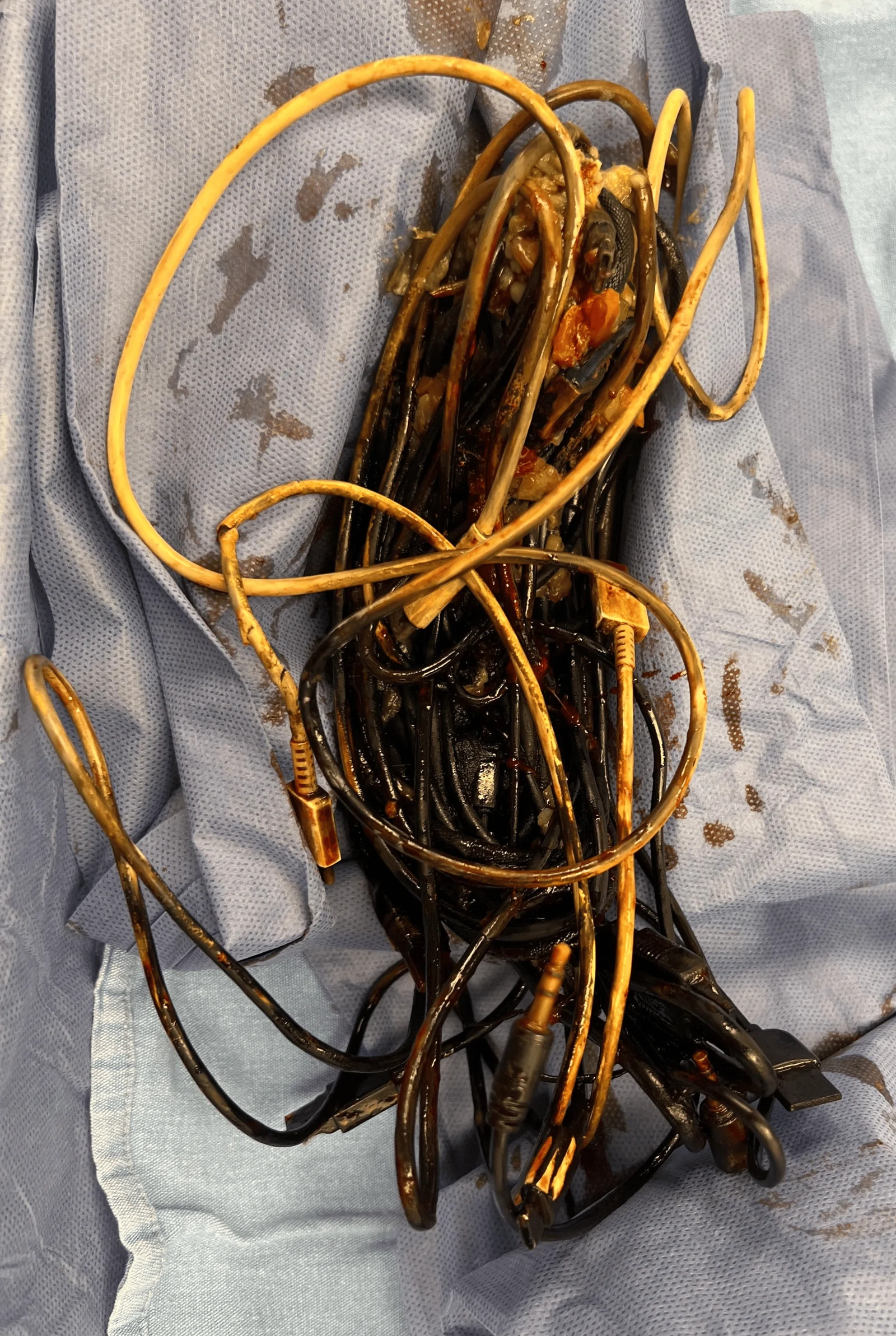
Macroscopic appearance of the extracted gastric cable bezoar Gross specimen after surgical extraction, demonstrating a compact conglomerate of seven intertwined cables with visible connectors and adherent intragastric debris. Most were mobile phone charging cables. The cables ranged approximately from 30 cm to 100 cm in individual length, with an estimated combined weight of approximately 200 g. These values represent retrospective gross estimates rather than formal calibrated measurements.

During the first postoperative day, the patient had mild abdominal discomfort and distension that resolved with continued recovery. He tolerated a liquid diet, passed flatus, and had no nausea or vomiting. The drain produced less than 50 mL of serohematic fluid during the first 24 hours and subsequently less than 20 mL of serous fluid by 48 hours. Oral intake was advanced to a complete diet. The operative wound remained well approximated without infection or dehiscence. The Blake drain was removed at discharge on postoperative day 2.

Psychiatry evaluated the patient during the hospitalization and established the diagnosis of pica, with outpatient psychiatric follow-up planned. At follow-up approximately one and two weeks after discharge, the patient continued to tolerate a normal diet without abdominal pain, nausea, or vomiting. The wound remained intact without infectious or dehiscence-related complications, and the skin staples were removed without difficulty. No early postoperative adverse event was reported.

A summary of the diagnostic, therapeutic, and follow-up course is provided in Table 2.

**Table 2:** Clinical timeline

Date	Clinical event
Initial presentation	Emergency evaluation for recurrence of an absence seizure; medical assessment initiated
During initial evaluation	A radiopaque foreign-body conglomerate was identified in the stomach; general surgery assumed care and completed preoperative assessment
Three days after presentation	Upper endoscopy demonstrated a dense network of cables occupying almost the entire gastric cavity; snare extraction was unsuccessful
Later the same evening	Open supraumbilical laparotomy, anterior gastrotomy, en bloc extraction of the cable bezoar, two-layer gastric closure, and Blake drain placement
Postoperative day 1	Early recovery with transient mild abdominal discomfort and distension, passage of flatus, and progression of oral intake
Postoperative day 2	Tolerating a complete diet; low-volume serous drain output; Blake drain removed and patient discharged home
1-2 weeks after discharge	No abdominal pain, nausea, vomiting, infection, or dehiscence; staples were removed without difficulty and recovery remained satisfactory

## Discussion

This case illustrates an unusual form of foreign-body gastric bezoar in which long, flexible charging cables became intertwined into a dense intragastric mass. Most bezoar literature focuses on plant fiber, hair, medications, or milk products [1,2]. By contrast, miscellaneous foreign-body bezoars arising from pica can have highly variable physical behavior, and their management is governed less by chemical composition than by bulk, rigidity, geometry, and the feasibility of safe transesophageal retrieval.

The association between pica and ASD is clinically important. Fields et al. found pica in 23.2% of preschool-aged children with ASD compared with 3.5% of population-based controls, although those pediatric prevalence estimates should not be directly extrapolated to adults [3]. The present patient had two major indicators of recurrence risk: a neurodevelopmental disorder with psychiatrist-confirmed pica and a prior laparotomy for ingestion of batteries and magnets seven years earlier. Recurrent foreign-body ingestion in neurologically or developmentally impaired patients has also been described in clinical series, where unwitnessed ingestion and multiple retained objects complicate diagnosis and management [6]. These features support a low threshold for considering occult ingestion when compatible imaging findings, unexplained gastrointestinal symptoms, or caregiver concerns arise.

Another notable feature was the paucity of gastrointestinal symptoms despite the volume of retained material. Foreign-body ingestion and bezoars may produce abdominal pain, nausea, vomiting, early satiety, bleeding, obstruction, or perforation, but asymptomatic or minimally symptomatic presentations are documented, particularly in patients in whom ingestion is unwitnessed or symptom reporting may be limited [1,7,8]. In this patient, the gastric mass was discovered incidentally during an admission initiated for recurrent absence seizures. Family members could only estimate that the ingestion might have occurred months earlier. The absence of peritoneal signs, obstructive symptoms, or free intraperitoneal gas suggested that the mass had remained confined to the stomach without an acute perforating complication at the time of diagnosis.

The radiographic appearance was unusually demonstrative. Because the ingested material contained radiopaque components, the plain abdominal radiograph outlined a mass of serpiginous linear densities that reproduced the gastric contour. The European Society of Gastrointestinal Endoscopy (ESGE) guidance supports plain radiography to establish the presence, location, number, size, and configuration of radiopaque or unknown ingested foreign bodies [4]. CT is recommended when perforation or another complication requiring surgery is suspected [4]. No abdominal CT was performed in this case; subsequent endoscopy directly defined the intragastric burden and demonstrated no duodenal extension.

Endoscopy was both diagnostic and initially therapeutic. It confirmed that the gastric lumen was nearly filled by a tightly interwoven cable network and demonstrated associated erythematous and erosive mucosal injury. Endoscopic retrieval is the preferred initial intervention for many upper gastrointestinal foreign bodies, but object dimensions and configuration determine feasibility [4,5]. The charging cables in this case behaved as a single mechanically complex mass rather than as independent retrievable objects. A metallic snare was used only to test mobilization; the mass remained completely fixed. Because the attempt never progressed to transesophageal withdrawal, no overtube or transparent hood was used. The inability to achieve even initial mobilization was treated as the safety threshold for abandoning further traction, thereby avoiding forceful manipulation that could increase the risk of mucosal injury or perforation.

This pattern resembles previously reported foreign-body bezoars in which endoscopic therapy failed and gastrotomy was required. Ahmed et al. described the surgical removal of numerous gastric foreign bodies after unsuccessful endoscopic retrieval [7]. Mulinder et al. reported rigid glove-derived esophageal and gastric bezoars in a patient with pica that were not amenable to endoscopic extraction and required laparotomy with gastrotomy [8]. In children and adolescents with ASD and pica, other unusual bezoar materials such as paper have also been reported; management has ranged from chemical dissolution to surgery depending on composition and mechanical characteristics [9]. A giant bezoar occupying the stomach and extending into the small bowel has likewise been described in a child with ASD, with surgical removal required after endoscopic confirmation [10]. These reports reinforce that "bezoar" is a morphologic concept rather than a single therapeutic entity: treatment must be individualized to the physical properties of the retained material.

The decision for open gastrotomy in our patient followed failed endoscopic extraction of a bulky, densely entangled mass occupying almost the entire stomach. The approximately 7-cm anterior gastrotomy allowed controlled en bloc removal, direct inspection of the lumen, and confirmation that no residual cable fragments remained. En bloc extraction was particularly advantageous because fragmentation could have produced multiple sharp or irregular segments, increased mucosal trauma, or left retained pieces. The uncomplicated postoperative course supports gastrotomy as a reliable definitive strategy when endoscopic removal is technically unsuccessful or judged unsafe.

Mucosal injury from chronic contact also deserves attention. The endoscopy demonstrated erythematous body and antral gastropathy together with superficial erosive pseudopolypoid antral lesions, without active bleeding. Bezoars may cause pressure-related mucosal injury, ulceration, and bleeding [1]. Other reports in patients with pica and ASD illustrate that gastric bezoars can produce complications beyond simple mechanical obstruction, including altered drug absorption and systemic toxicity [11]. Thus, even an apparently asymptomatic bezoar cannot be assumed to be biologically inert.

The current case also differs from reports of heterogeneous material ingestion. Mixed-material bezoars have been documented in patients with pica and ASD, including synchronous gastric and jejunal involvement [12]. In contrast, the present bezoar was strikingly dominated by one modern household object category, mobile phone charging cables, whose length, flexibility, polymer coating, and metallic components favored progressive entanglement and produced a radiographically recognizable gastric cast. To our knowledge, published reports specifically centered on a gastric bezoar predominantly composed of intertwined mobile phone charging cables are extremely limited. The value of the case therefore lies not only in the unusual material and the complete radiographic-endoscopic-operative correlation but also in the strikingly paucisymptomatic presentation, with incidental detection of a large gastric foreign-body burden in a patient who continued to tolerate oral intake without clinical or radiographic evidence of gastrointestinal obstruction.

Definitive treatment should address recurrence risk in addition to removing the intragastric mass. The prior ingestion requiring laparotomy, the newly established diagnosis of pica, and the current need for repeat surgery identify this patient as being at substantial risk for future episodes. Psychiatric and behavioral follow-up, caregiver education, environmental control of accessible ingestible objects, and prompt assessment of suspected recurrent ingestion are therefore important components of longitudinal care. The patient was referred for outpatient psychiatric follow-up after discharge.

The principal limitation of this report is that formal calibrated measurement and scale-based weighing of the extracted material were not performed at the time of surgery. Retrospective gross assessment identified seven cables, with approximate individual lengths ranging from 30 cm to 100 cm and an estimated combined weight of approximately 200 g; therefore, these quantitative values should be interpreted as clinical estimates rather than formal specimen measurements. The aggregate cable length was not recorded. The time of ingestion also could not be established precisely because the episode was unwitnessed. Nevertheless, the diagnosis and extent of disease are supported by concordant radiographic, endoscopic, and operative documentation, and complete early follow-up was available through two weeks.

## Conclusions

Foreign-body bezoars should remain in the differential diagnosis of patients with pica and neurodevelopmental disorders even when gastrointestinal symptoms are minimal or absent. Long, flexible objects such as charging cables can accumulate silently, intertwine, and form a mechanically complex gastric mass that is readily visible on radiography but difficult to remove endoscopically.

When endoscopic extraction is unsuccessful because of bulk or entanglement, gastrotomy provides definitive access for controlled en bloc removal and direct confirmation of gastric clearance. Equally important, surgical treatment should be coupled with psychiatric and behavioral follow-up aimed at preventing recurrence, particularly in patients with a prior history of clinically significant foreign-body ingestion.
